# Expanding the clinicopathological‐genetic spectrum of GNE myopathy by a Chinese neuromuscular centre

**DOI:** 10.1111/jcmm.16978

**Published:** 2021-10-22

**Authors:** Kai‐Yue Zhang, Hui‐Qian Duan, Qiu‐Xiang Li, Yue‐Bei Luo, Fang‐Fang Bi, Kun Huang, Huan Yang

**Affiliations:** ^1^ Department of Neurology Xiangya Hospital Central South University Changsha China; ^2^ Clinic Medicine of 8‐year Program Xiangya School of Medicine Central South University Changsha China; ^3^ Institute of Molecular Precision Medicine and Hunan Key Laboratory of Molecular Precision Medicine Xiangya Hospital, Central South University Changsha China

**Keywords:** *GNE* mutation, GNE myopathy, muscle pathology, myopathy, neuromuscular disorder

## Abstract

GNE myopathy is a heterogeneous group of ultrarare neuromuscular disorders caused by mutations in the *GNE* gene. An estimated prevalence of 1~21/1,000,000 leads to a deficiency of data and a lack of availability of samples to conduct clinical research on this neuromuscular disorder. Although *GNE*, which is the mutated gene responsible for the disease, is well known as the key enzyme in the biosynthesis pathway of sialic acid, the clinicopathological‐genetic spectrum of *GNE* mutant patients is still unclear and expanding. This study presents ten unrelated patients with GNE myopathy, discovering five novel missense mutations. Clinical, electrophysiological, imaging, pathological and genetic data are presented in a retrospective manner. Interestingly, several patients in the cohort were found to have peripheral neuropathy and inflammatory cell infiltration in muscle biopsies, which have seldom been reported. This study, conducted by a neuromuscular centre in China, is the first attempt to highlight these abnormal clinicopathological features and associate them with genetic mutations in GNE myopathy.

## 1 INTRODUCTION

GNE myopathy, also known as distal myopathy with rimmed vacuoles (DMRV), hereditary inclusion body myopathy (HIBM) or inclusion body myopathy 2 (IBM2), was first reported in Japanese patients by Nonaka in 1981.^1^ It is a rare, recessively inherited muscle disease caused by mutations in the *GNE* gene (9p13.3) encoding the bifunctional enzyme UDP‐N‐acetylglucosamine (GlcNAc) 2‐epimerase/N‐acetylmannosamine (ManNAc) kinase, a significant rate‐limiting enzyme of the sialic acid biosynthesis pathway.^2^ GNE myopathy is clinically characterized by progressive weakness and atrophy of distal lower‐limb muscles that preferentially involve the tibialis anterior muscles and spare the quadriceps,^3^ with normal or mildly increased serum creatine kinase (CK) levels.^4^ Peripheral neuropathy is not a typical presentation but can be seen in several cases. Pathological features of GNE myopathy include specific rimmed vacuoles, muscle fibre atrophy and a muscle volume decrease. Notably, inflammatory infiltrations are rarely seen in GNE myopathy, different from sporadic inclusion body myositis (sIBM), but without any satisfactory explanation for the clinical presentation.

To date, more than 201 *GNE* mutations associated with GNE myopathy have been reported,^5^ with missense mutations making up a clear majority. The exact pathomechanism of GNE myopathy is still unknown but is most likely attributable to aberrant protein sialylation, identified as a common result of decreased GNE enzyme activity. Currently, a definite diagnosis of GNE myopathy mainly relies on genetic testing, confirmed by evidence of compound heterozygous or homozygous mutations in the *GNE* gene.^6^ Different *GNE* mutations have been detected worldwide, and they present with different prevalences in populations of diverse ethnicities, such as c.2228T>C (p.M743T) in the Middle East, c.1807G>C (p.V603L) and c.620A>T (p.D207V) in Japan and c.2179G>A (p.V727 M) in South‐East Asia.^7^, ^8^ Meanwhile, patients with different *GNE* variants experience varying ages of onset and other clinical features, suggesting that different variants have different functional impacts.

In this study, we described the clinicopathological and genetic profiles of ten Chinese patients with GNE myopathy, among which five novel mutations were found, broadening the mutation spectrum of the *GNE* gene. In addition, we analysed the presence of two relatively rare clinicopathological manifestations in GNE myopathy, peripheral neuropathy and muscle inflammation, and summarized the genotype‐phenotype correlations of the *GNE* mutations.

## 2 MATERIALS AND METHODS

### 2.1 Ethics approval

Ethics approval was granted by the Ethics Committee of Xiangya Hospital, Central South University. Informed consent for participation in our research was obtained from all of the patients, as previously reported in our centre.^9^

### 2.2 Patients and clinical evaluation

From 2014 to 2021, ten patients were diagnosed with GNE myopathy based on clinical manifestations, pathological findings and genetic testing in the neuromuscular centre of Xiangya Hospital, Central South University. Clinical assessment of the patients consisted of a physical examination and laboratory investigations, such as serum creatine kinase (CK), electromyography (EMG), muscle biopsy, magnetic resonance imaging (MRI) of the thigh and leg muscles, and genetic testing, as previously used in our centre.^10^

### 2.3 Biopsies and pathological examination

Muscle biopsies were obtained from the tibialis anterior or biceps brachii muscles. Nerve biopsies were performed on the sural nerves. Pathological examination was performed as described elsewhere with minor modifications.^11^, ^12^ First, the samples were frozen in isopentane cooled with liquid nitrogen and cut into 5 μm thick sections using a cryostat. The sections were stained with haematoxylin and eosin (HE), modified Gömöri trichrome, acid phosphatase, nicotinamide adenine dinucleotide (NADH), succinic dehydrogenase (SDH), cytochrome C oxidase, adenosine triphosphatase (ATPase) (Ph: 4.2, 4.6 and 9.6), periodic acid‐Schiff (PAS) and oil red O (ORO).

### 2.4 Genetic analysis

Genomic DNA (gDNA) was extracted from peripheral blood (MyGenostics) using a DNeasy Blood and Tissue Kit (Qiagen, Venlo) as previously mentioned^13^, ^14^ according to the manufacturer's instructions. Next‐generation sequencing (NGS) analysis covering 2082 genes known to be associated with neuromuscular disorders was performed. The sequences obtained were compared with those in the human genome database. Functional prediction software, polymorphism Phenotyping version 2 (PolyPhen‐2, http://genetics.bwh.harvard.edu/pph2/) and Mutation Taster (http://www.mutationtaster.org/) were used to predict the possible impact of the identified substitution on protein structure and function.

## 3 RESULTS

### 3.1 Clinical characteristics

In our current research, ten patients diagnosed with GNE myopathy were recruited. The cohort of patients showed a female predominance with a male to female ratio of 3:7. The age of disease onset ranged from 20.0 to 43.0 years (median, interquartile range: 28.5 [21.5–32.5] years), and the disease duration ranged from 0.5 to 10.0 years (median, interquartile range: 6.0 [2.8–7.8] years), respectively. At the first consultation, eight patients (80.0%) had muscle weakness of the limbs, one patient (10.0%) had atrophy of the lower distal muscles, and the other patient (10.0%) had muscle weakness of the waist and backache. All patients underwent a muscular strength assessment. In addition to the involvement of the limb muscles, varying degrees of weakness of the neck and waist were demonstrated in nine patients (90.0%) and six patients (60.0%), respectively. None of the patients complained of cardiac or respiratory problems. Notably, all cases (100.0%) presented with obvious muscle atrophy. Among them, four patients (40.0%) had both upper and lower distal limb atrophy, three patients (30.0%) had only lower‐limb atrophy, two patients (20.0%) exhibited a pattern of mixed proximal and distal limb atrophy, and the last patient (10.0%) had all limbs involved. Only two patients (20.0%) had limb numbness. Tendon reflexes were decreased or even disappeared in half of the patients (50.0%) who were Gowers’ sign positive. The clinical features and muscular strength assessment are summarized in Tables 1 and 2. The echocardiography and electrocardiogram were performed in all the patients to measure the cardiac comorbidities, but none of the patients were found abnormal.

**TABLE 1:** Clinical features of the ten patients with GNE myopathy

Patient No.	Gender	Onset age (years)	Assessment age (years)	Duration (years)	Initial symptoms	Numbness	Muscular atrophy	Tendon reflex	Gowers’ sign	Ambulation at assessment
1	F	28	38	10	Atrophy of LDL	−	UDL+LDL	Decreased	+	Ambulant
2	F	20	20	0.5	Muscle weakness of BLL	−	UDL+BLL	Normal	−	WCD
3	F	29	31	2	Muscle weakness and numbness of AL	AL	UDL+LDL	Normal	−	Ambulant
4	F	33	41	8	Muscle weakness of BLL	−	AL (with quadriceps)	Normal	−	Ambulant
5	F	23	33	10	Muscle weakness of AL	−	UDL+LDL	Decreased	+	Ambulant
6	F	43	49	6	Muscle weakness of AL	UPL−left	UPL+LDL	Decreased	+	Ambulant
7	M	38	45	7	Backache and muscle weakness of waist	−	BLL	Disappeared	+	Ambulant
8	M	20	22	2	Muscle weakness of BLL	−	LDL	Normal	−	Ambulant
9	M	31	37	6	Muscle weakness of AL	−	UPL+LDL	Disappeared	+	Ambulant
10	F	21	26	5	Muscle weakness of BLL	−	LDL	Normal	+	Ambulant

Abbreviations: AL, all limbs; BLL, both lower limbs; F, female; LDL, lower distal limbs; LPL, lower proximal limbs; M, male; UDL, upper distal limbs; UPL, upper proximal limbs; WCD, wheelchair‐dependent.

**TABLE 2:** Muscular Strength (MRC grade) of the patients

Patient No.	UPL	UDL	LPL	LDL	scapular	cervical	Iliopsoas
1	4	3	4	3	4	3	3
2	5‐	4	4	3	5	4	5
3	5	5‐	5‐	4‐	5	5‐	5
4	4	3	4	3‐	4	2	3
5	4	4‐	4	2	5‐	4	4
6	5	4	5	4	4	4	4‐
7	4+	L5R4	L3R4	3	5‐	3	3
8	4+	4	4	3	4	4	4+
9	3+	5	4	4	3+	2	2
10	5‐	5	5	2+	5	3	5

Abbreviations: L, left; LDL, lower distal limbs;LPL, lower proximal limbs; MRC: Medical Research Council; R, right; UDL, upper distal limbs; UPL, upper proximal limbs.

**FIGURE 1:**
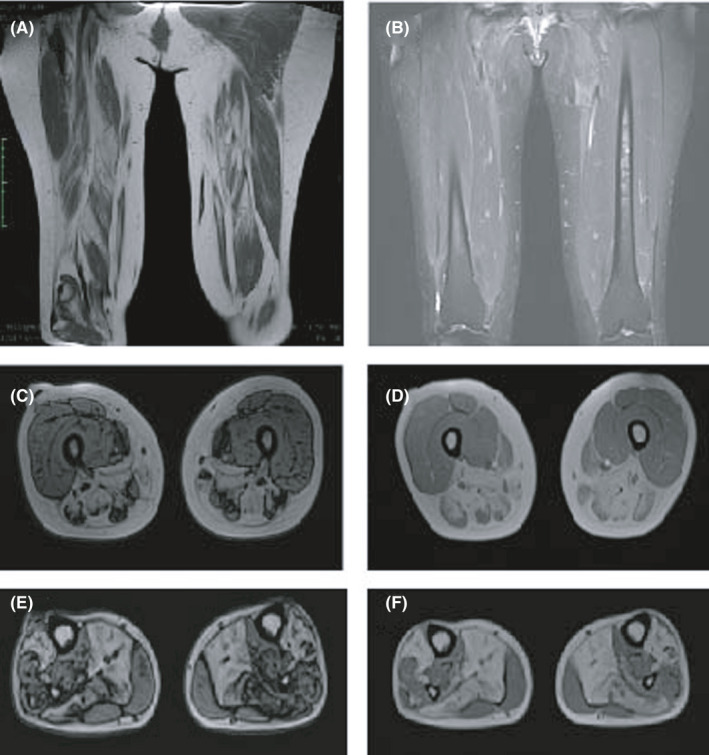
MRI in patient No.5 revealed increased signal intensity of the femur shaft and hamstrings; MRI in patient No.6 revealed pronounced fatty infiltration along with muscle atrophy in the posterior and internal compartments of the thigh muscles and lower leg muscles. (A and B), coronal axial MRI image of thigh muscles in patient No.5. (C and D), transverse axial image of thigh muscles in patient No.6. (E and F), transverse axial image of lower leg muscles in patient No.6

Except for the abnormally high value of 1277.0 U/L of patient No.8, the level of CK ranged from 165.3 to 542.1 U/L (median, interquartile range: 265.9 [243.5–354.6] U/L, reference value: 40.0–200.0 U/L). Electromyography (EMG) analysis revealed simple myopathic changes in only half of the patients (50.0%), including abnormal spontaneous potentials, early recruitment and a pattern of small amplitude, short duration and increased percentage of polyphasic waves of motor unit potentials (MUPs). Another four patients (40.0%) showed myopathic damage accompanied with neuropathic lesion, including fibrillation potentials, positive sharp waves and decreased motor nerve conduction velocity. One patient (10.0%) showed prolonged duration and normal amplitude MUPs with a vast of denervated potentials, suggesting impaired peripheral nerves injury of distal upper and lower limbs. Generally, the impairment found by EMG was predominant in distal limb muscles. More details of the EMG examination are shown in Table 3. MRI was performed for two patients (20.0%), showing extensively increased signal intensity of the femur shaft and hamstrings in patient No.5 and serious fatty infiltration along with muscle atrophy in patient No.6 (Figure 1).

**TABLE 3:** Details of examinations and follow‐up of the patients

Patient No.	CK level (U/L)^a^	EMG	MRI	Muscle biopsy	Nerve biopsy	Treatment	Follow‐up
1	353.9	M/N (decreased MCV)	NA	IFSV + A + R + I + LP		Levocarnitine (2.0 g/d)	PD
2	282.8	N	NA	IFSV + A + D + R + HC + IN		Vitamin B1 (60.0 mg/d)	PD
3	243.1	M/N (decreased MCV)	NA	IFSV + D + R + HC + ME + HM	Swelling and loss of myelinated fibre; axonal degeneration	Vitamin B1 (60.0 mg/d), Mecobalamin (1.5 mg/d)	PD
4	165.3	M	NA	IFSV + A + D + R + HC + HM + IN + LP		Adenosine disodium triphosphate (60.0 mg/d)	SD
5	244.7	M	Multiple abnormal signals in femoral shaft and extensively increased signals in left hamstrings	IFSV + R + HC + ME		Levocarnitine (2.0 g/d), adenosine Disodium triphosphate (60.0 mg/d)	PD
6	249.0	M/N	Fatty infiltration and muscle atrophy	IFSV + I + LP		None	NA
7	354.8	M	NA	IFSV + A + D + R + I		Levocarnitine (2.0 g/d)	PD
8	1277.0	M	Diffuse oedema and swelling of multiple calf muscles	IFSV + D + R + HC		Adenosine disodium triphosphate (60.0 mg/d), Vitamin B1 (60.0 mg/d)	NA
9	229.1	M	NA	IFSV + R + HC + HM + LP		Adenosine disodium triphosphate (60.0 mg/d)	PD
10	542.1	M/N	NA	IFSV + A + D + R + I + HC + HM + LP		None	NA

Abbreviations: M, myogenic lesion; N, neurogenic lesion; M/N, myogenic lesion accompanied with neurogenic lesion; MCV, motor nerve conduction velocity; NA, not available; IFSV, increased fibre size variation; A, atrophic myofibre; D, degenerative, necrotic and regenerative myofibre; R, rimmed vacuoles; I, inflammatory infiltration; HC, hyperplasia of connective tissue; ME, moth‐eaten myofibre; HM, hypertrophic myofibre; IN, internal nuclei; LP, lipid droplets; PD, progressive disease; SD, stable disease; None, refusal to any treatment.

^a^ Reference value of CK: 40.0–200.0 U/L.

### 3.2 Muscle and nerve pathological features

**FIGURE 2:**
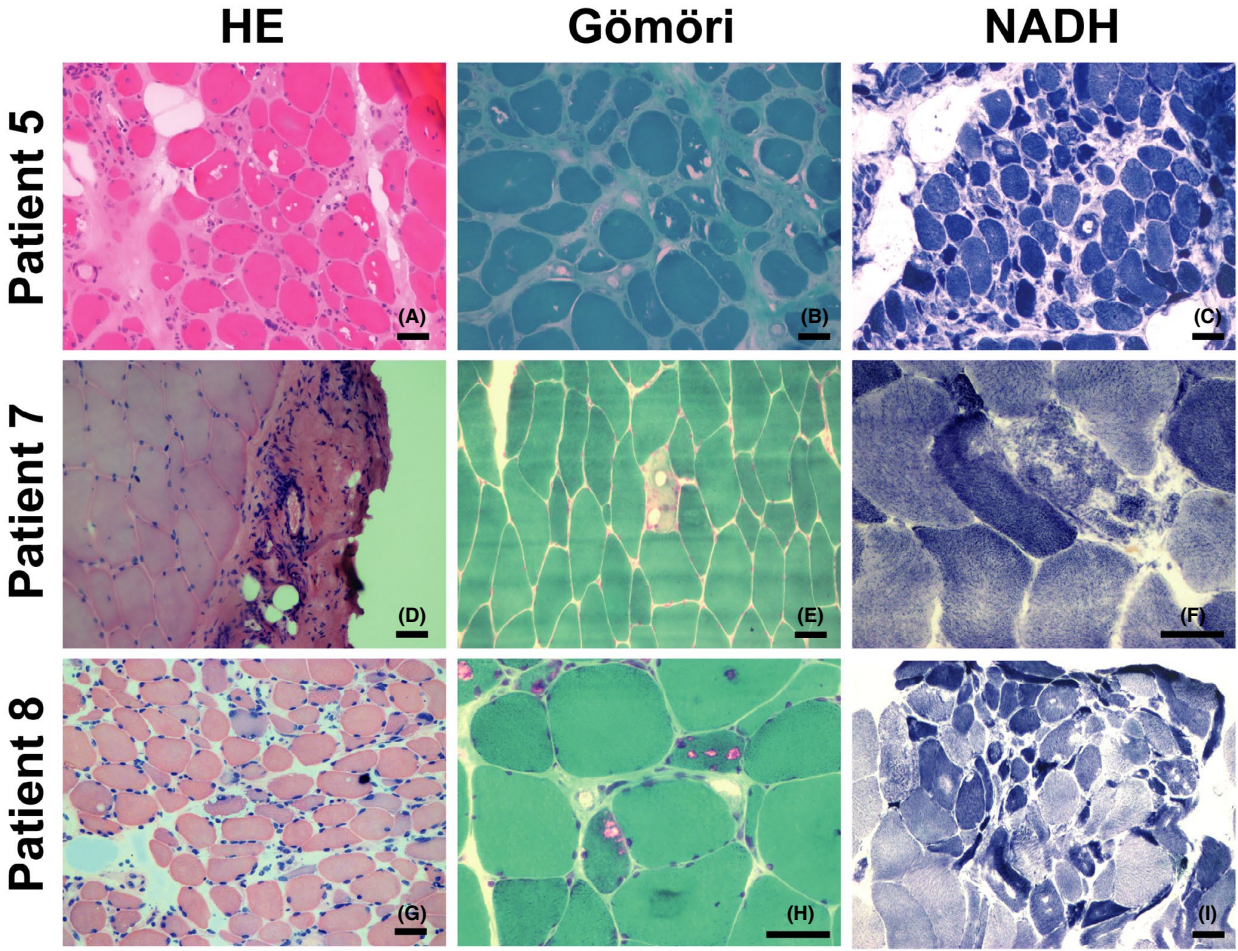
Myopathological changes in patients with GNE myopathy. All specimens were obtained from skeletal muscle. HE staining showed increased fibre size variation (A, G), vacuoles (A), fascial inflammation (D), atrophy (A, G) and regeneration (G). Modified Gömöri trichrome staining showed vacuoles (B) and rimmed vacuoles (E, H) in the fibres. NADH staining showed cores (C, I) and myofibrillar network disarray (F). Scale bar = 50 μm

Muscle biopsies were performed for all the patients, and nerve biopsies were conducted in one patient. Increased fibre‐size variation, rimmed vacuoles and internal nuclei were the most common pathologic changes in nearly all of the muscle samples (Figure 2). Degeneration, necrosis and moderate‐to‐severe hyperplasia of the connective tissue were detected in six patients (60.0%). Lipid droplets in the muscle cells were found in half of the patients (50.0%). NADH staining revealed moth‐eaten myofibres in two patients (20.0%) and myofibrillar disarrays in five patients (50.0%). Slight inflammation was found in four patients (40.0%). Hypertrophic myofibre and fibre splitting were found in four patients (40.0%). Acid phosphatase staining revealed increased enzyme activity and glycogen granules in the necrotic muscle fibres.

**FIGURE 3:**
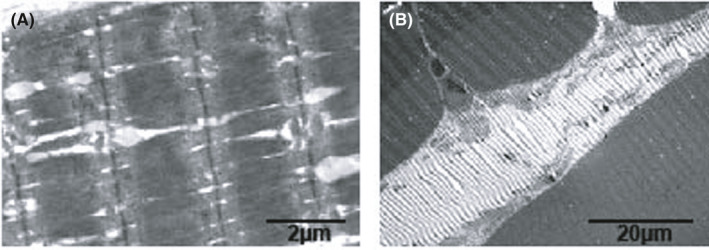
Myopathological changes under electron microscopy in patient No.1 showed atrophic muscle fibres, a dilated sarcoplasmic reticulum and some oedematous and vacuolated mitochondria

By electron microscopy, the atrophic muscle fibres appeared to be small and irregular in shape. The sarcoplasmic reticulum was dilated, and the mitochondria were oedematous and vacuolated (Figure 3). A sural nerve biopsy performed in patient No.3 showed that myelinated fibres were mildly decreased in number. Furthermore, oedema and degeneration of the myelin sheath and axon were also confirmed by electron microscopy. The results of the muscle biopsy are shown in Table 3.

### 3.3 Genetic analysis

NGS analysis covering 2082 genes that have been confirmed to have a link with neuromuscular disorders was conducted. In our cohort, seven patients (70.0%) were confirmed to carry compound heterozygous mutations, while three patients (30.0%) had homozygous missense mutations. In sum, twelve mutations of the *GNE* gene were detected, among which c.830G>A (p.R277Q), c.1985C>T (p.A662V), c.620A>T (p.D207V), c.125G>A (p.R42Q), c.1616T>C (p.L539S), c.C577T (p.R193C) and c.C124T (p.R42W) have been previously described as pathogenic mutations of GNE myopathy.^15^, ^16^ Interestingly, three hotspot mutations, c.125G>A (p.R42Q), c.620A>T (p.D207V) and c.830G>A (p.R277Q), appeared repeatedly, and the latter two have been described in previous studies as the most common pathogenic mutations in Chinese patients.^17^, ^18^ Besides the mutations in *GNE*, other heterozygous mutations were also detected in patients No.3, 5, 6, 7 and 8, but all these gene‐related diseases are recessive diseases, and we defined these variants are benign (Table S1–S5).

To our knowledge, this is the first report of two novel heterozygous missense mutations, c.2099G>A (p.G700E) and c.539C>T (p.A180V), and three homozygous missense mutations, c.1489A>G (p.R497G), c.959A>G (p.Q320R) and c.854A>G (p.D285G), causing GNE myopathy (Table 4). Subsequently, we screened hundreds of alleles from normal Chinese individuals, and we did not identify any of these genetic changes. We think that these five novel mutations are likely to be pathogenic based on the predictions of PolyPhen‐2 and MutationTaster. The predicted scores and results of the functional prediction software programs are shown in Table 5.

**TABLE 4:** Gene Mutation of the ten patients with GNE myopathy

No.	Zygosity	*GNE* mutations							
		Allele 1	Exon	Reported	Family member (with the same mutation)	Allele 2	Exon	Reported	Family member (with the same mutation)
1	Het	c.577C>T (p.R193C)	3	Y	F	c.124C>T (p.R42W)	2	Y	M/S
2	Het	c.830G>A (p.R277Q)	4	Y	M	c.539C>T (p.A180V)	3	N	F
3	Het	c.830G>A (p.R277Q)	4	Y	F	c.2099G>A (p.G700E)	12	N	M
4	Het	c.620A>T (p.D207V)	3	Y	F	c.125G>A (p.R42Q)	2	Y	M/S
5	Hom	c.1489A>G (p.R497G)	8	N	M/F	–	–	–	–
6	Het	c.620A>T (p.D207V)	3	Y	Unknown	c.1616T>C (p.L539S)	9	Y	Unknown
7	Het	c.620A>T (p.D207V)	3	Y	Unknown	c.125G>A (p.R42Q)	2	Y	Unknown
8	Het	c.830G>A (p.R277Q)	4	Y	Unknown	c.1985C>T (p.A662V)	11	Y	Unknown
9	Hom	c.959A>G (p.Q320R)	6	N	M/F	–	–	–	–
10	Hom	c.854A>G (p.D285G)	5	N	M/F	–	–	–	–

Abbreviations: Het, heterozygous; Hom, homozygous; Y, Yes; N, No; F, father; M, mother; M/F, both father and mother; S, son.

**TABLE 5:** Predicted results of five novel mutations in the *GNE* gene from several functional prediction software programs

	c.539C>T (p.A180V)		c.1489A>G (p.R497G)		c.2099G>A (p.G700E)		c.959A>G (p.Q320R)		c.854A>G (p.D285G)	
	Score	Prediction	Score	Prediction	Score	Prediction	Score	Prediction	Score	Prediction
PolyPhen−2	0.979	Probably damaging	0.975	Probably damaging	1.000	Probably damaging	0.085	Benign	0.183	Benign
Mutation Taster	0.999	Disease causing	0.999	Disease causing	0.999	Disease causing	0.999	Disease causing	0.999	Disease causing

## 4 DISCUSSION

Patients with different *GNE* variants have varying ages of onset and other clinical features, suggesting that different variants have diverse functional impacts that are critical to consider in disease interventions and prognostication.^8^ The understanding of GNE myopathy is limited due to the rarity of GNE myopathy per se. For a long time, it was assumed that the characteristic hallmarks of GNE myopathy were substantial rimmed vacuoles predominantly in atrophic fibres, while the presence of inflammation in muscle biopsies should be excluded from the diagnostic criteria. This is reminiscent of the unusual manifestation of peripheral neuropathy in GNE myopathy, which was not given enough attention in previous studies. This is the first report to highlight these atypical symptoms and to demonstrate a possible correlation between GNE genotype and phenotype.

Two of the most frequent mutations in our patients, c.620A>T (p.D207V) and c.830G>A (p.R277Q), which might be hotspot mutations for the *GNE* gene in China, have been studied before.^7^, ^18^ Previous studies have revealed that c.620A>T (p.D207V) is the most common mutation in Chinese patients and is the second most common mutation in Japan. Chen's^7^ study compared their GNE myopathy patient groups carrying c.620A>T (p.D207V) in the epimerase domain with patients carrying other mutations and found that the patients carrying c.620A>T (p.D207V) tended to show a late onset (median, interquartile range: 31.0 [24.8–38.2] vs 25.0 [22.0–30.8] years, *p* < 0.001), which is in concordance with our results (median, interquartile range: 38.0 [35.5–40.5] years vs 23.0 [20.5–28.5] years, *p* < 0.05). To further investigate the genotype‐phenotype correlations, we searched for additional articles describing cases with c.620A>T (p.D207V) mutation and listed four of them with comprehensive information about their clinical and pathological features in Table 6.^19^, ^20^

**TABLE 6:** Clinical features and examinations of previously reported cases with significant *GNE* mutations

Mutation	No.	Gender	Onset age (year)	Initial symptom	Muscle weakness	Quadriceps sparing	Numbness	Muscular atrophy	CK level (U/L)	EMG	Muscle biopsy
c.620>T (p.D207V)	Ia	F	29	Muscle weakness of BLL	BLL + IP	+	–	–	258.0	M	F + A + D + R
	Ib	M	43	Muscle weakness of AL	UDL + BLL	+	–	UDL + LDL	578.0	M	F + A + D + R + I
	Ic	F	34	Muscle weakness of BLL	UDL + BLL + IP	–	–	UDL + LDL	254.0	M	F + A + D + R
	II	M	29	Muscle weakness of BLL	LDL	+	–	–	1621.0	M	F + A + D + R
c.830G>A (p.R277Q)	IIIa	F	18	Muscle weakness of BLL	AL	+	–	NM	284.0	M	A + R
	IIIb	F	27	Muscle weakness of BLL	AL	–	–	NM	NM	M	NM
	IIIc	M	27	Muscle weakness of BLL	UDL + BLL	+	–	NM	NM	M	NM
	IIId	F	28	Muscle weakness of LDL	UDL + BLL	+	–	NM	172.0	M	A + D + R + IN
	IV	F	21	Muscle weakness of LDL	AL	–	–	NM	294.0	M	F + R
	V	M	18	Muscle weakness of LDL	UPL + LDL	+	–	NM	384.0	NM	NM
	VI	NM	18–24	Muscle weakness of LDL	NM	+	–	NM	NM	NM	NM
	VII	F	21	Muscle weakness of LDL	AL	+	–	NM	NM	N	R
	VIII	F	19–34	Muscle weakness of LDL	NM	Unknown	–	NM	NM	NM	NM

Abbreviations: UPL, upper proximal limbs; UDL, upper distal limbs; LPL, lower proximal limbs; LDL, lower distal limbs; IP, iliopsoas; BLL, both lower limbs; AL, all limbs; NM, not mentioned; M, myogenic lesion; N, neurogenic lesion; F, fibre size variation; A, atrophic myofibre; D, degenerative, necrotic and regenerative myofibre; R, rimmed vacuoles; I, inflammatory infiltration; IN, internal nuclei.

In our cohort, three patients were found to carry c.830G>A (p.R277Q) heterozygous mutation. They all presented in their early twenties and had relatively severe lower‐limb weakness, especially of the distal muscles. In comparison with patients with c.620A>T (p.D207V) mutation, the muscles of the shoulder, neck and waist were mildly or not involved; however, nerve impairment tended to be much more common, accounting for 2/3 patients in our cohort. Another four cases with c.830G>A (p.R277Q) homozygous mutation and five cases with heterozygous variants reported in previous studies are listed in Table 6.^15^, ^21^, ^22^ Notably, the median onset age of the patients who were homozygous (median, interquartile range: 27.0 [20.2–27.8] years) was approximately six years later than the others (median, interquartile range: 21.0 [19.5–23.8] years), which was similar to our patients harbouring the same heterozygous variant (median, interquartile range: 20.0 [20.0–24.5] years). Except for the absence of sensory symptoms and cardiac involvement, the clinical features were similar to our cases. Quadriceps weakness was observed in one patient with a homozygous mutation and in three patients in a heterozygous state, suggesting a possible link between genotype and phenotype. In addition to c.830G>A (p.R277Q) mentioned above, another variant, c.829C>T (p.R277W), has also been reported in previous articles.^23^

Mutations of c.1616T>C (p.L539S) and c.1985C>T (p.A662V) have previously been reported in patients of varying ethnicity, including Japanese, Jewish, Scottish and Chinese patients.^4^, ^15^, ^17^, ^23^, ^24^ A British^24^ study revealed that patients with c.1616T>C (p.L539S) variant first showed symptoms on average 7.2 years earlier than those without this mutation.

In our study, two different mutations, c.124C>T (p.R42W) (patient No.1) and c.125G>A (p.R42Q) (patient No.4/No.7), caused a replacement of arginine with different amino acids in the same position of the *GNE* gene; the former was substituted by tryptophan, whereas the latter was substituted by glutamine. Three patients exhibited typical muscle weakness, with wider coverage and remarkable involvement of the upper limbs than the others without this mutation. On muscle biopsy, in addition to some characteristic changes, inflammatory infiltration was also observed between the muscle fibres and fascia in two patients (2/3), which did not frequently appear in other cases of GNE myopathy. To our knowledge, muscle weakness is always the most common first symptom of GNE myopathy, while atrophy gradually develops during the later course of disease.^25^ It is worth mentioning that patient No.1 first presented with simple muscle atrophy in appearance of the lower distal limbs, without functional weakness or numbness. It is still uncertain whether the difference in primary symptoms is associated with c.124C>T (p.R42W) mutation.

In recent years, due to the increased availability of genetic testing, a growing number of cases with *GNE* mutations have been reported. Surprisingly, some patients also showed a notable association with peripheral neuropathy.^26^ In our study, nearly half of the patients presented with myopathic lesions accompanied by neuropathic changes during the progression of the disease, suggesting potential nerve involvement in the pathogenesis of GNE myopathy. The specific aetiology of neuropathy complications remains unknown. In a previous study,^27^ a significant reduction in the mRNA and protein levels of peroxiredoxin IV was observed in *GNE* mutant (c.620A>T (p.D207V) and c.1807G>C (p.V603L)) cells. Interestingly, peroxiredoxin IV acts as an important ER‐resident H_2_O_2_ sensor in cells to regulate neurogenesis, which means that its downregulation may not only affect the ER redox state but also inhibit nerve development. Although peripheral neuropathy is not regarded as a remarkable clinical manifestation of GNE myopathy, it is probably underestimated and exists as a sign of disease deterioration.

The spectrum of diseases caused by *GNE* mutations is constantly growing. Interestingly, no patient has been identified carrying biallelic nonsense mutations or frameshifting mutations thus far,^15^ suggesting that some basic activity of GNE is required during embryonic or early development. In mice, the GNE protein is expressed and plays an important role in an early embryonic stage, and *Gne* ^−/−^ is lethal to mice, which is consistent with the clinical lack of biallelic null mutations and only ‘mildly deleterious’ mutations reported in GNE myopathy patients.^28^ Recently, NGS has become more widely available, leading to an increasing understanding not only of GNE myopathy‐related mutations, but also of other myopathies.^29^

At present, there is no effective therapy available for GNE myopathy.^30^, ^31^ Preclinical studies have identified that the use of oral monosaccharides reversed muscle hyposialylation in a GNE myopathy mouse model.^32^ However, phase II and III randomized studies evaluating sialic acid extended‐release for GNE myopathy showed two distinct results; the phase II study was positive for the curative effect of N‐acetylneuraminic acid (Ace‐ER), while the latter study showed no improvement of muscle strength after Ace‐ER intake compared with placebo.^33^ Additional studies are urgently needed to identify an effective treatment for GNE myopathy.

## CONFLICT OF INTEREST

The authors declare no conflict of interest.

## AUTHOR CONTRIBUTIONS

**Kai‐Yue Zhang:** Investigation (equal); Methodology (equal); Software (equal); Validation (equal); Writing‐original draft (lead); Writing‐review & editing (lead). **Hui‐Qian Duan:** Investigation (equal); Resources (equal). **Qiu‐Xiang Li:** Formal analysis (equal); Resources (equal). **Yue‐Bei Luo:** Formal analysis (equal); Methodology (equal); Software (equal). **Fang‐Fang Bi:** Investigation (equal); Writing‐review & editing (equal). **Kun Huang:** Conceptualization (equal); Supervision (equal); Writing‐review & editing (equal). **Huan Yang:** Conceptualization (equal); Project administration (lead); Supervision (equal).

## Supporting information

Appendix S1

## Data Availability

The original data that described in this study are available from the corresponding author upon reasonable request.
