# Supplementary material for: Expanding the clinicopathological‐genetic spectrum of GNE myopathy by a Chinese neuromuscular centre
Source: J Cell Mol Med. 2021 Oct 22;25(22):10494–503. doi: 10.1111/jcmm.16978 (PMC8581342; doi:10.1111/jcmm.16978)
Supplement: Supplementary file 1 — Appendix S1 [file JCMM-25-10494-s001.docx]

**Expanding the clinicopathological-genetic spectrum of GNE myopathy by a Chinese neuromuscular centre**

Kai-Yue Zhang^1,2^, Hui-Qian Duan^1^, Qiu-Xiang Li^1^, Yue-Bei Luo^1^, Fang-Fang Bi^1^, Kun Huang^1,3,*^, Huan Yang^1,*^

*^1^ Department of Neurology, Xiangya Hospital, Central South University, Changsha, Hunan province, China*

*^2^ Clinic Medicine of 8-year Program, Xiangya School of Medicine, Central South University, Changsha, Hunan province, China*

*^3^ Institute of Molecular Precision Medicine and Hunan Key Laboratory of Molecular Precision Medicine, Xiangya Hospital, Central South University, Changsha, Hunan province, China.*

***^*^ Correspondence:***

*Kun Huang, MD, PhD, Department of Neurology, Institute of Molecular Precision Medicine and Hunan Key Laboratory of Molecular Precision Medicine, Xiangya Hospital, Central South University, Xiangya Road, Kaifu District, Changsha 410008, China. Email: huangkn@outlook.com.*

*or Huan Yang, MD, PhD, Department of Neurology, Xiangya Hospital, Central South University, Xiangya Road, Kaifu District, Changsha 410008, China. Email: yangh69@126.com.*

Supplementary information includes:

Supplementary Table S1-S5.

**Supplementary Table S1** Other mutations of case 3

| Gene | OMIN number | Genotype | cDNA | Protein | Related disease |
| --- | --- | --- | --- | --- | --- |
| *PNPLA2* | 610717 | AR | c.118G>A | p.A40T | neutral lipid storage myopathy |
| *PRX* | 614895 | AR | c.2968C>T | p.D990N | Charcot-Marie-Tooth disease, demyelinating, type 4F |

**Supplementary Table S2** Other mutations of case 5

| Gene | OMIN number | Genotype | cDNA | Protein | Related disease |
| --- | --- | --- | --- | --- | --- |
| *SMPD1* | 607608 | AR | c.995C>G | p.P332R | Niemann-Pick disease, type A/ type B |
| *FREM1* | 608944 | AR/AD | c.767A>C | p.N256T | Bifid nose with or without anorectal and renal anomalies/ Manitoba oculotrichoanal syndrome/ Trigonocephaly 2 |

**Supplementary Table S3** Other mutations of case 6

| Gene | OMIN number | Genotype | cDNA | Protein | Related disease |
| --- | --- | --- | --- | --- | --- |
| *ABCA3* | 601615 | AR | c.5020G>A | p.G1674S | Surfactant metabolism dysfunction, pulmonary, 3 |
| *AGRN* | 103320 | AR | c.1352G>A | p.R451H | Myasthenic syndrome, congenital, 8, with pre- and postsynaptic defects |
| *ANK3* | 600465 | AR | c.1825G>A | p.D609N | Mental retardation, autosomal recessive, 37 |
| *ANK3* | 600465 | AR | c.2722G>A | p.G908R | Mental retardation, autosomal recessive, 37 |
| *CLCNKB* | 602023 | AR | complete gene duplication | － | Bartter syndrome, type 3 |
| *COL18A1* | 120328 | AR | c.1264C>T | p.R422C | Knobloch syndrome, type 1 |
| *DNAH5* | 603335 | AR | c.1089+1G>A | － | Ciliary dyskinesia, primary, 3, with or without situs inversus |
| *DPYD* | 612779 | AR | c.220C>T | p.R74* | Dihydropyrimidine dehydrogenase deficiency |
| *FANCD2* | 613984 | AR | c.2783delC | p.L928Rfs*14 | Fanconi anemia, complementation group D2 |
| *HSPG2* | 142461 | AR | c.7438C>T | p.R2480W | Dyssegmental dysplasia, Silverman-Handmaker type/ Schwartz-Jampel syndrome, type 1 |
| *LCT* | 603202 | AR | c.82T>G | p.F28V | Lactase deficiency, congenital |
| *NDUFA2* | 602137 | AR | c.191A>G | p.K64R | Mitochondrial complex I deficiency, nuclear type 13 |
| *NDUFS1* | 157655 | AR | c.1285G>A | p.V429M | Mitochondrial complex I deficiency, nuclear type 5 |
| *SGCA* | 600119 | AR | c.320C>T | p.A107V | Muscular dystrophy, limb-girdle, autosomal recessive 3 |
| *SLC25A13* | 603859 | AR | c.2T>C | p.M1? | Citrullinemia, adult-onset type II/ Citrullinemia, type II, neonatal-onset |

**Supplementary Table S4** Other mutations of case 7

| Gene | OMIN number | Genotype | cDNA | Protein | Related disease |
| --- | --- | --- | --- | --- | --- |
| *DYSF* | 603009 | AR | c.2864+7C>A | － | Miyoshi muscular dystrophy 1/ Muscular dystrophy, limb-girdle, autosomal recessive 2/ Myopathy, distal, with anterior tibial onset |

**Supplementary Table S5** Other mutations of case 8

| Gene | OMIN number | Genotype | cDNA | Protein | Related disease |
| --- | --- | --- | --- | --- | --- |
| *ASPM* | 605481 | AR | c.9466C>T | p.Q3156* | Microcephaly 5, primary, autosomal recessive |
| *LONP1* | 605490 | AR | c.2282C>T | p.P761L | CODAS syndrome |
| *NPHP1* | 607100 | AR | complete gene duplication | － | Joubert syndrome 4/ Nephronophthisis 1, juvenile/ Senior-Loken syndrome-1 |
